# Absent in melanoma 2 enhances anti‐tumour effects of CAIX promotor controlled conditionally replicative adenovirus in renal cancer

**DOI:** 10.1111/jcmm.15697

**Published:** 2020-07-29

**Authors:** Dafei Chai, Dong Qiu, Zichun Zhang, Shang Yuchen Shi, Gang Wang, Lin Fang, Huizhong Li, Hailong Li, Hui Tian, Junnian Zheng

**Affiliations:** ^1^ Cancer Institute Xuzhou Medical University Xuzhou China; ^2^ Center of Clinical Oncology Affiliated Hospital of Xuzhou Medical University Xuzhou Medical University Xuzhou China; ^3^ Department of Urology Affiliated Hospital of Xuzhou Medical University Xuzhou Medical University Xuzhou China; ^4^ Department of Radiation Oncology Affiliated Hospital of Xuzhou Medical University Xuzhou Medical University Xuzhou China

**Keywords:** AIM2, CAIX promotor, CRAds, inflammasome, renal cancer, treatment

## Abstract

Conditionally replicative adenoviruses (CRAds) were promising approach for solid tumour treatment, but its oncolytic efficiency and toxicity are still not satisfactory for further clinical application. Here, we developed the CAIX promotor (CAIX^promotor^)‐controlled CRAd armed with a tumour suppressor absent in melanoma 2 (AIM2) to enhance its oncolytic potency. The CAIX^promotor^‐AIM2 adenoviruses (Ad‐CAIX^promotor^‐AIM2) could efficiently express E1A and AIM2 in renal cancer cells. Compared with Ad‐CAIX^promotor^, Ad‐CAIX^promotor^‐AIM2 significantly inhibited cell proliferation and enhanced cell apoptosis and cell killing, thus resulting in the oncolytic efficiency in 786‐O cells or OSRC‐2 cells. To explore the therapeutic effect, various Ads were intratumourally injected into OSRC‐2‐xenograft mice. The tumour growth was remarkably inhibited in Ad‐CAIX^promotor^‐AIM2‐treated group as demonstrated by reduced tumour volume and weight with a low toxicity. The inflammasome inhibitor YVAD‐CMK resulted in the reduction of anti‐tumour activity by Ad‐CAIX^promotor^‐AIM2 in vitro or in vivo, suggesting that inflammasome activation response was required for the enhanced therapeutic efficiency. Furthermore, lung metastasis of renal cancer mice was also suppressed by Ad‐CAIX^promotor^‐AIM2 treatment accompanied by the decreased tumour fossil in lung tissues. These results indicated that the tumour‐specific Ad‐CAIX^promotor^‐AIM2 could be applied for human renal cancer therapy. The therapeutic strategy of AIM2‐based CRAds could be a potential and promising approach for the therapy of primary solid or metastasis tumours.

## 1 INTRODUCTION

Conditionally replicative adenoviruses (CRAds) were dependent on tumour‐specific promotors by the control of virus gene (such as E1A) expression for selective replication.^1^, ^2^ The direct consequence of Ad replication results in tumour cell killing.^3^ In contrast to other agents for current cancer therapies, CRAds possess the advantages of conditional selective replication viral self‐spreading, highly transduction efficacy and subsequently augment anti‐tumour effects in cancer cells.^4^, ^5^ Many attempts of CRAds have been made great development in cancer gene therapies, but treatment of solid or metastasis tumours remains a challenge.^6^, ^7^, ^8^ Thus, novel therapeutic strategies for cancer should be developed to enhance the oncolytic efficiency with a low toxicity.

Tumour‐specific promotors can control the expression of viral genes or therapeutic genes to select replication in tumour cells instead of normal cells.^9^, ^10^ The cell surface protein, carbonic anhydrase IX (CAIX), is a tumour‐sassociated antigen of renal cancer.^11^ CAIX expression is almost not detected on normal kidney cells or other normal cells, but an increasing expression is detected on 90% of renal cancer lesions.^12^ CAIX antigen shows apparent tumour specificity in renal cancer, and is involved in tumour cell proliferation, oncogenesis and tumour progression.^13^, ^14^ Therefore, CAIX promotor (CAIX^promotor^) could serve as an ideal target to construct tumour‐specific Ad for renal cancer treatment. To increase the safety of Ad, some modifications have been introduced to restrict Ad replication to tumour cells.^15^ We have previously constructed a novel oncolytic Ad with E1A gene controlled by CAIX^promotor^.^1^ This modification is to replace promotors for essential viral genes with specific promotor that perform activation only in tumour cells.^16^

Ads assembled with therapeutic genes can amplify the capable of lysing tumour cells selectively.^2^, ^17^ Absent in melanoma 2 (AIM2) is a member of the interferon‐inducible HIN‐200 protein family, and is associated with inflammation and cancer pathology.^18^, ^19^ AIM2 as a DNA sensor can form inflammasome complex by binding to cytosolic DNA and subsequently activate caspase‐1 to produce IL‐1β for regulating inflammatory responses.^20^ Of note, AIM2 as a tumour suppressor is a prognostic marker and an independent predictor of disease progression in renal cell cancer, and is also used as the therapy target for renal cancer therapy.^21^ Previous studies demonstrated that overexpression of AIM2 can exert anti‐tumoural activities through enhancing the inflammasome activation to induce cell apoptosis or autophagy.^21^, ^22^ Thus, AIM2 might be applied as a therapeutic gene to enhance the potential efficacy of CARd for renal cancer treatment.

In the present study, we constructed CAIX^promotor^ controlled oncolytic adenovirus expressing AIM2 (Ad‐CAIX^promotor^‐AIM2) and evaluated its efficacy and toxicity in renal cancer cells or tumour models. Ad‐CAIX^promotor^‐AIM2 decreased cell viability, and induced cell apoptosis and cell killing in human renal cancer cells. Additionally, Ad‐CAIX^promotor^‐AIM2 displayed potent anti‐tumour activity in OSRC‐2‐xenograft model or lung metastasis model. Our results indicated that the oncolytic Ad carrying a tumour therapeutic gene might be effective and safe for primary solid or metastasis tumour therapy.

## 2 MATERIALS AND METHODS

### 2.1 Animals

4‐6 weeks old male nude mice (BALB/c‐nu/nu) were purchased from Vital River Laboratory and maintained under pathogen‐free conditions. All procedures for animal experiments were approved by the Committee on the Use and Care of Animals and performed in accordance with the guidelines of the Laboratory Animal Ethical Committee of Xuzhou Medical University.

### 2.2 Cell lines and cell culture

Normal renal tubular epithelial cell line HK‐2, human renal cancer cell line 786‐O, OSRC, ACHN and Kert‐3, and human embryonic kidney 293 cell line HEK293 were purchased from the Chinese Academy of Sciences (Shanghai, China). All cells were authenticated by short tandem repeat profiling or certificate of analysis. These cells were cultured in Dulbecco's Modified Eagle Medium (DMEM, Gibco, Thermo Fisher Scientific) supplemented with 10% foetal bovine serum (FBS, ExCell Bio, China) and maintained in 37°C in a 5% CO2 humidified incubator. All experiments were performed with mycoplasma‐free cells.

### 2.3 Plasmid and virus preparation

pZD55, pZD55‐CAIX^promotor^, pCA13, pcDNA3‐FLAG3C‐AIM2 and pBHGE3 plasmids were previously stored or constructed in our laboratory. The AIM2 expression cassette excised from pcDNA3‐FLAG3C‐AIM2 was firstly subcloned into pCA13 to form pCA13‐AIM2. The expression cassette containing the AIM2 controlled by the human CMV promotor was then digested with BglII and subcloned into the pZD55 (for pZD55‐AIM2) or pZD55‐CAIX^promotor^ (for pZD55‐CAIX^promotor^‐AIM2) successfully. The Ad pZD55 (Ad‐Ctrl), Ad pZD55‐AIM2 (Ad‐AIM2), Ad pZD55‐CAIX^promotor^ (Ad‐CAIX^promotor^) and Ad pZD55‐CAIX^promotor^‐AIM2 (Ad‐CAIX^promotor^‐AIM2) were generated by co‐transfecting with packaging plasmid pBHGE3 in HEK293 cells. Large‐scale Ads were purified from the supernatant of infected cells by ultracentrifugation at 50,000g for 2 h at 4°C. The titres were determined by plaque assays on HEK293 cells.

### 2.4 Flow cytometry analysis

For determining human CAIX expression, cell samples were stained with anti‐human PE‐conjugated anti‐CAIX (R&D Systems) and incubated at 4°C for 30 minutes. The labelled cells were then washed with PBS and data acquisition was performed. For E1A or AIM2 expression determination, cells were fixed and permeabilized for with cytofix/cytoperm (BD Biosciences) 30 minutes, washed two times with permeabilization wash buffer (BD Biosciences) and incubated with mouse anti‐E1A antibody (Merck Millipore) or mouse anti‐AIM2 antibody (Santa Cruz) at 4°C for overnight. The labelled cells were then washed with permeabilization wash buffer and stained with Alexa647‐conjugated goat anti‐mouse lgG (Vicman, China), then stained cells were washed, and data acquisition was carried out on BD FACSCanto II (BD Biosciences) in FACSDiva software (BD Biosciences) and analysed by FlowJo software (Tree Star Inc).

### 2.5 Cytotoxicity assay

Cells were seeded at a density of 3 × 10^5^ per well in a six‐well culture plate. After 24 h, cells were infected with Ad‐Ctrl, Ad‐AIM2, Ad‐CAIX^promotor^ and Ad‐CAIX^promotor^‐AIM2, respectively, at various MOIs: 0, 0.1, 1, 10 and 100. Five days after infection, the cells were exposed to 2% crystal violet in 20% methanol for 15 min, washed with distilled water (ddH_2_O) and photographed.

### 2.6 Cell proliferation assay

Cells were seeded at a density of 1 × 10^4^ per well in a 96‐well culture plate. After 24 h, cells were infected with various Ads, respectively, at a 10 MOI. Cell proliferation was detected at 24, 48 and 72 h after infection. 10 μl solution of Cell Counting Kit‐8 (CCK‐8, Beyotime, China) was added to each well and incubated at 37°C for 1 h. Absorbance at 450 nm was measured on an ELX‐800 spectrometer reader (Bio‐Tek Instruments).

### 2.7 Colony formation assay

Cells were trypsinized, counted and seeded in 6‐well plate at a density of 1 × 10^3^ cells per well. After 24 h, cells were infected with various Ads, respectively, at a 10 MOI. After 7 days of incubation, survival colonies were fixed with 4% paraformaldehyde for 10 minutes and stained with 0.5% of crystal violet and counted under the microscope.

### 2.8 Cell apoptosis detection

The treated cells were collected and stained with an Annexin V‐FITC/PI apoptosis detection kit (BD Biosciences) according to the manufacturer's instructions. Cell apoptosis was analysed by flow cytometry.

### 2.9 Real‐time PCR

Total RNA were extracted from collected cells by Trizol (Invitrogen) and reverse‐transcribed (RT) using a cDNA synthesis kit (TaKaRa, China) according to the manufacturer's instructions. The expression of the genes encoding AIM2, ASC, caspase‐1 and IL‐1β was quantified by real‐time PCR using 7900HT qPCR system thermal cycler (Applied Biosystems) and SYBR Green system (TaKaRa) normalized by GAPDH expression following the manufacturer's protocol. Primers were used as following in Table 1.

**Table 1:** Primers were used as follow

	Forward primer	Reverse primer
GAPDH	ATCCCATCACCATCTTCCAG	GAGTCCTTCCACGATACCAA
AIM2	GGCCCAGCAGGAATCTATCA	GCTTGCCTTCTTGGGTCTCA
ASC	CTTATCGCGAGGGTCACAAAC	CCTTGCAGGTCCAGTTCCA
Caspase‐1	CCTCAGGCTCAGAAGGGAATG	CGTGTGCGGCTTGACTTGT
IL‐1β	ACGAATCTCCGACCACCACTA	GGAAAGAAGGTGCTCAGGTCAT

### 2.10 Caspase‐1 activity assay

Caspase‐1 activity was detected by Caspase‐1 Activity Assay Kit (Solarbio Biotech) according to the manufacturer's instructions. Briefly, for cell samples, 2‐10 × 10^6^ cells were lysed in 50‐100 μl lysis buffer on ice for 10 min. For tissue samples, 3‐10 mg tissues were added to 100 μl lysis and were homogenated with a tissue homogenizer. The supernatant from extracts was collected by centrifuged at 12 000 g for 10 min. Protein concentrations were detected using the Bradford method, ensuring that the protein concentration was 1‐3 μg/μl. A standard curve was prepared using the pNA standard. 10‐35 μl volume of supernatant was incubated with 5 μl 2 mM Ac‐YVAD‐pNA substrate at 37°C. Then, the absorbance values of pNA were read on a spectrophotometer at the optical density 405nm (OD_405_). The changes of caspase‐1 activity were calculated by the radio of OD_405_ of the experimental wells to that of the control wells.

### 2.11 ELISA measurement of cytokines

To assess protein levels of IL‐1β, IL‐6 concentrations in culture supernatant or in homogenized tumour tissues of mice, enzyme‐linked immunosorbent assays (ELISA) were performed according to the manufacturer's instructions (eBioscience).

### 2.12 Animal model

To establish human renal cancer subcutaneous xenograft mouse model, OSRC‐2 cells were harvested and resuspended in Matrigel which was diluted with RPMI‐1640 medium in 1:1 ratio to a density of 1 × 10^7^ cells/mL. The resuspended cells (200 μl/mouse) were subcutaneously injected into the hind legs of nude mice. When tumours reached approximately 80‐120 mm^3^ in volume, the mice were randomly divided into four groups (six mice per group) and treated with intratumour injections of Ad‐Ctrl, Ad‐AIM2, Ad‐CAIX^promotor^ and Ad‐CAIX^promoter^‐AIM2 at a dose of 2 × 10^8^ plaque forming units (PFU) per mouse every other day, with a total dosage of 1 × 10^9^ pfu of Ads. The survival status of tumour‐bearing mice was monitored daily. The tumour diameters were measured twice a week. Tumour volume was calculated using the following equation: Volume= (A × B^2^)/2 (A refers to the long diameter and B refers to the short diameter). Animals were killed and tumour tissues were surgically excised from the mice. Tumour weight was evaluated.

In metastasis model, mice were intravenously injected with 4 × 10^6^ OSRC‐2 cells from the tail vein at day 0. Mice were divided into four groups and injected with various Ads at a dose of 4 × 10^8^ PFU/ mouse every other day, with a total dosage of 2 × 10^9^ pfu of Ads. 28 days after tumour inoculation, mice were killed and lungs were removed, and the metastatic nodules were quantified.

### 2.13 Pathological analyses

For routine histological analyses, murine tissues were surgically resected and fixed in 4% paraformaldehyde (Sigma‐Aldrich), embedded in paraffin and cut into sections 5 μm sections. H&E staining was performed according to the manufacturer's instructions, and pictures were acquired with microscope (Nikon). The numbers of metastatic nodules or the quantification of metastatic foci were assessed in the sections by a pathologist blinded to treatment group.

### 2.14 Statistical analysis

Statistical analysis was performed with GraphPad Prism (Version 5.01) software and expressed as means ± SD. Statistical significance was evaluated using two‐tailed Student's t test. Multiple comparisons were performed using one‐way ANOVA. The statistical significance level was set as *P < .05; **P < .01; and ***P < .001.

## 3 RESULTS

### 3.1 Construction and preparation of Ad‐CAIX^promotor^‐AIM2

**Figure 1:**
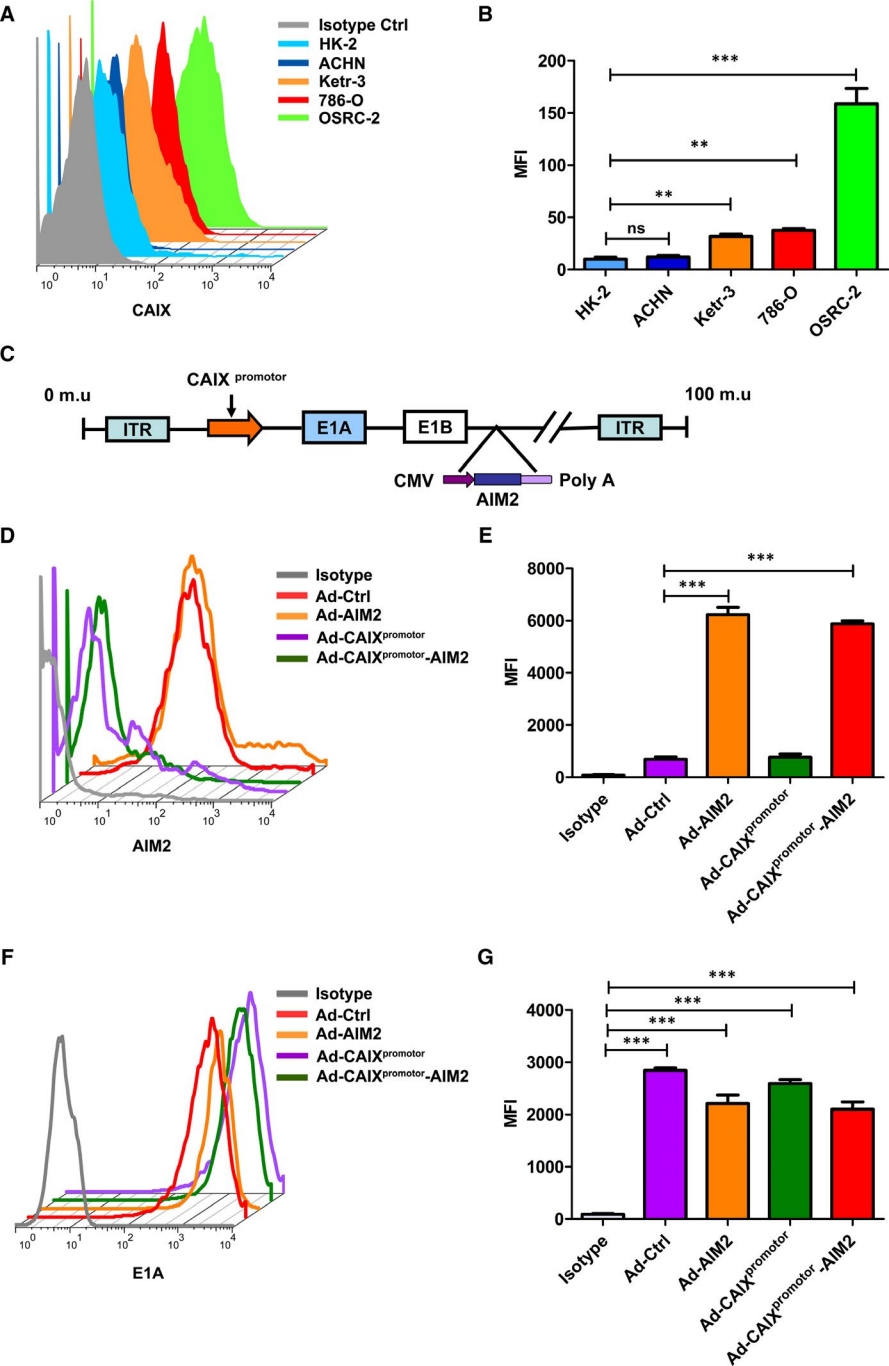
The construction and expression of Ad‐CAIX^promotor^‐AIM2. (A) The expression levels of CAIX were detected by flow cytometry in HK‐2, ACHN 786‐O, Ketr‐3 and OSCR‐2 cells. (B) The data shown as statistical analysis of MFI in (A). (C) The schematic structure of Ad‐CAIX^promotor^‐AIM2. (D) AIM2 expression was evaluated in OSRC‐2 cell line by flow cytometry. (E) The values of MFI were estimated in (D). (F) The expression level of E1A was detected by flowcytometry in OSCR‐2 cells. (G) The statistical analysis of MFI in (F). Data represent the means of three independent experiments. Data are shown as means ± SD. The different significance was set at *P < .05, **P < .01 and ***P < .001

**Figure 2:**
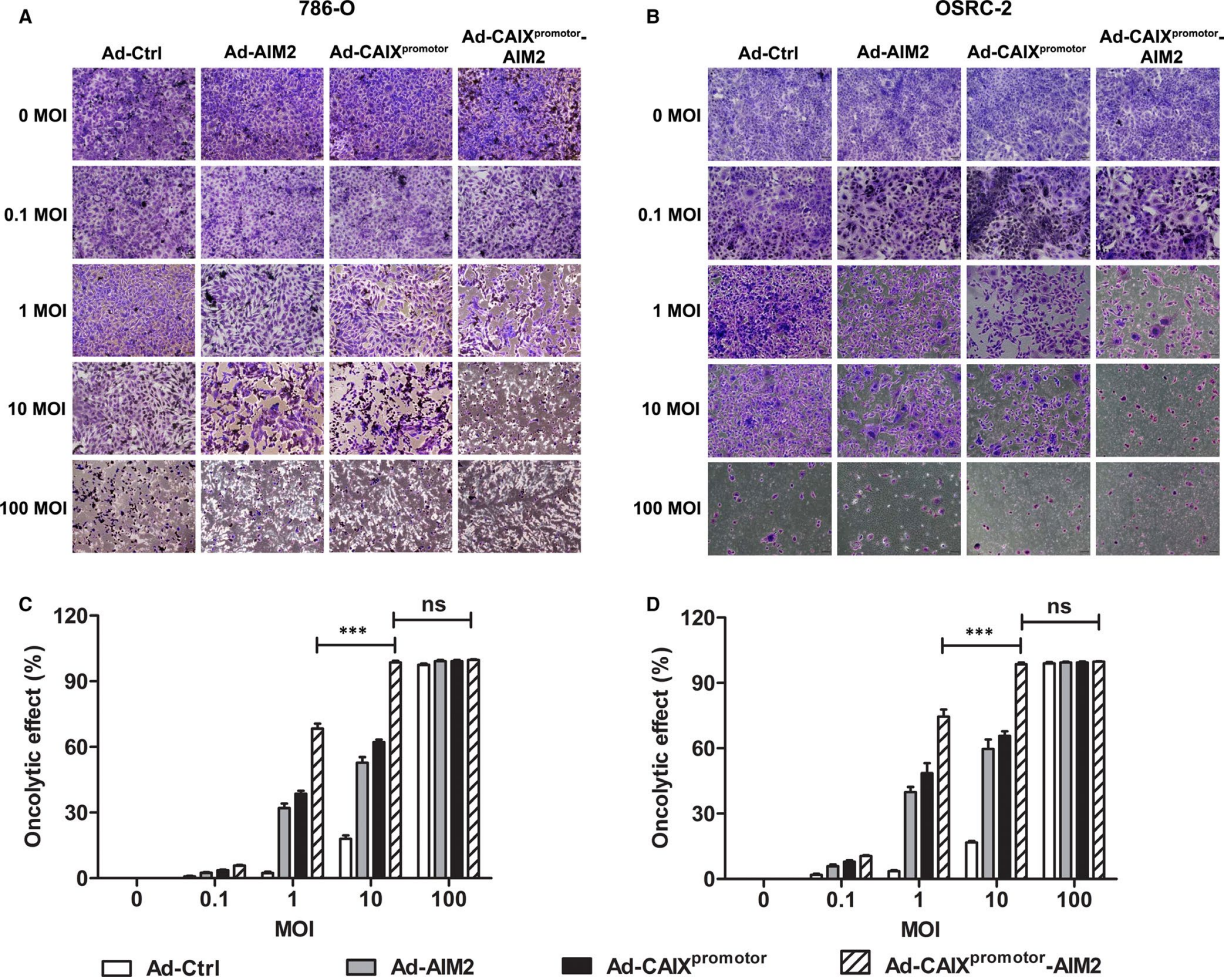
Tumour‐selective cytopathic effect of Ad‐CAIX^promotor^‐AIM2. 786‐O and OSRC‐2 cells were seeded in 24‐well plates at a density of 1 × 10^4^ cells for each well and infected with Ad‐Ctrl, Ad‐AIM2, Ad‐CAIX^promotor^ or Ad‐CAIX^promotor^‐AIM2 at the indicated multiplicities of infection (MOIs). Seven days later, cells were stained with crystal violet. (A and B) The crystal violet staining of 786‐O cells or OSRC‐2 cells infected with Ads. (C and D) The oncolytic effect was calculated in (A) or (B). Data are from one representative experiment of three performed and presented as the mean ± SD. The different significance was set at *P < .05, **P < .01 and ***P < .001; ns, not significant

To determine CAIX expression in HK‐2, ACHN, Ketr‐3, 786‐O and OSRC‐2, these cell lines were examined by flow cytometry with CAIX staining antibody. Compared with that in normal human cells (HK‐2), human renal cancer cell lines Ketr‐3, 786‐O and OSRC‐2 showed highly positive CAIX expression, and in ACHN it was very low (Figure 1A,B). CAIX^promotor^ could serve as an ideal target to construct tumour‐specific adenovirus for renal cancer treatment. Therefore, a novel CAIX^promotor^ controlled oncolytic adenovirus was constructed by introduced the therapeutic gene of AIM2 for enhancing its oncolytic potency (Figure 2C). For evaluation of Ad‐CAIX^promotor^‐AIM2 for cancer‐specific expression, E1A or AIM2 expression was detected by flow cytometry in OSRC‐2 cells infected with Ad‐Ctrl, Ad‐AIM2, Ad‐CAIX^promotor^ or Ad‐CAIX^promotor^‐AIM2, respectively. As shown in Figure 1D,E, the MFI analysis showed that a significant increasing expression of AIM2 was observed in Ad‐AIM2 or Ad‐CAIX^promotor^‐AIM2‐infected cells compared with the control cells, and indicated AIM2 gene was successfully expressed in Ad‐AIM2 or Ad‐CAIX^promotor^‐AIM2‐infected renal cancer cells. E1A is a prerequisite for Ad replication and an indicator of the potential of an adenovirus to lyse infected cells.^23^ Accordingly, E1A protein confirmed by the MFI also could be efficiently expressed in Ad‐CAIX^promotor^ or Ad‐CAIX^promotor^‐AIM2‐infected renal cancer cells (Figure 1F,G), and indicated Ad‐CAIX^promotor^ or Ad‐CAIX^promotor^‐AIM2 efficiently droved the E1A expression in CAIX antigen‐positive cells. These results indicated that Ad‐CAIX^promotor^‐AIM2 had a higher selectivity in CAIX antigen‐positive renal cancer cell lines.

### 3.2 Ad‐CAIX^promotor^‐AIM2 enhanced the oncolytic efficiency in renal cancer cells

To evaluate the oncolytic efficiency of Ad‐CAIX^promotor^‐AIM2, the cytotoxic effects of oncolytic Ads on 786‐O or OSRC cells were investigated by crystal violet staining. Cells were infected with Ad‐Ctrl, Ad‐AIM2, Ad‐CAIX^promotor^ or Ad‐CAIX^promotor^‐AIM2 at a MOI of 0, 0.1, 1, 10 and 100. Five days later, cells were stained with crystal violet. As shown in Figure 2, 786‐O or OSRC cells were completely killed infected with each virus at a MOI of 100. The infection of Ad‐CAIX^promotor^‐AIM2 at a MOI of 10 also killed the renal cancer cells. At a MOI of 10, Ad‐AIM2 and Ad‐CAIX^promotor^ could only kill them partially (Figure 2A‐D).

**Figure 3:**
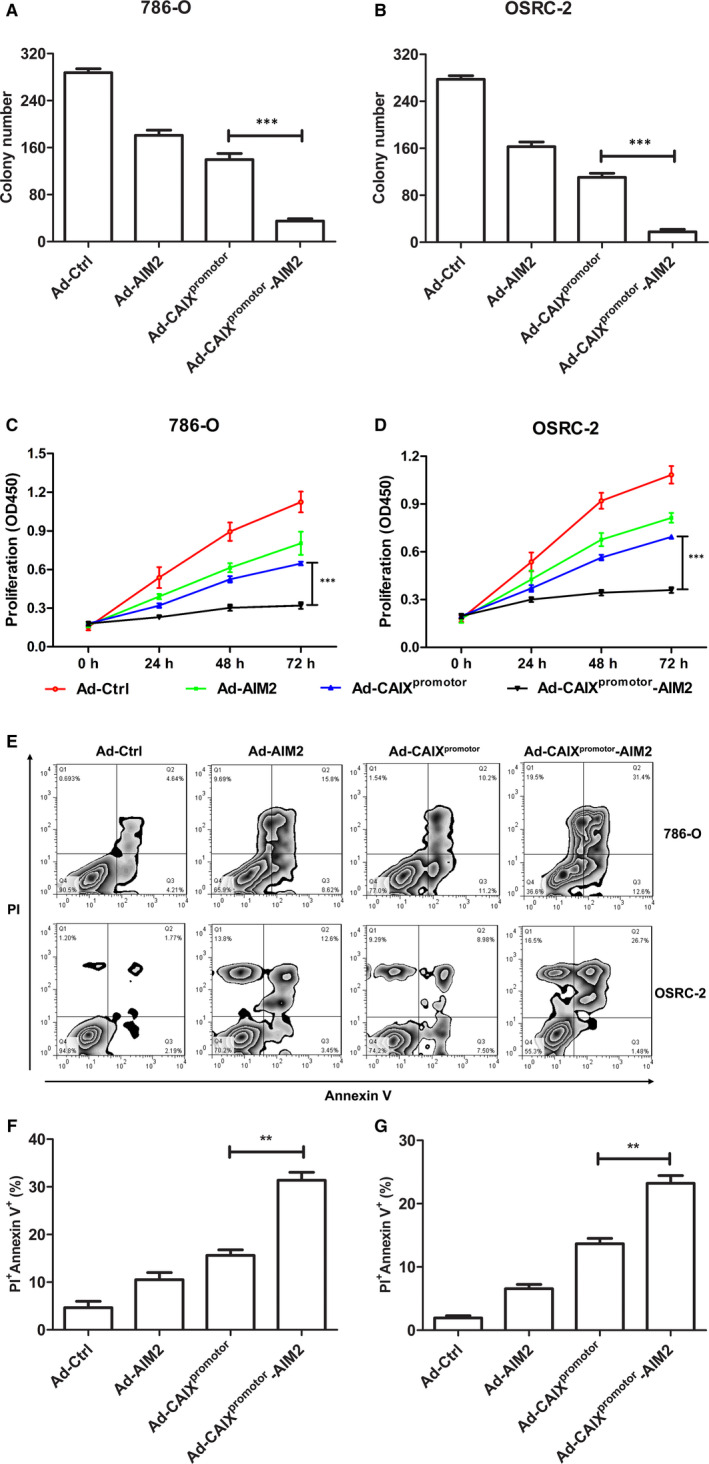
Ad‐CAIX^promotor^‐AIM2 enhanced the inhibition of cell proliferation and promoted cell apoptosis. (A and B) The colony formation capability of Ad‐CAIX^promotor^‐AIM2 or control‐treated 786‐O and OSRC‐2 were detected at day 7. (C and D) The cell proliferation was detected by CCK‐8 assay in Ad‐CAIX^promotor^‐AIM2 or control‐treated renal cancer cells. (E) Forty‐eight hours after Ad‐CAIX^promotor^‐AIM2 or control infection, the cell apoptosis was detected by flow cytometry in both cell lines. (F and G) The percentages of apoptotic cells were evaluated in (E). All experiments were carried out in triplicate. Data are shown as means ± SD. The different significance was set at *P < .05, **P < .01 and ***P < .001

Effect of Ad‐CAIX^promotor^‐AIM2 on cell proliferation in renal cancer cells was assessed by the colony formation assay in vitro. The results indicated that a significant inhibition effect was observed on 786‐O cells or OSRC cells infected with Ad‐CAIX^promotor^‐AIM2 compared with Ad‐CAIX^promotor^ (Figure 3A,B). CCK‐8 assays demonstrated that Ad‐CAIX^promotor^‐AIM2 remarkably reduced the proliferation of renal carcinoma cells at time‐dependent (Figure 3C,D), and this reduced proliferation was stronger on OSRC‐2 cells than that in 786‐O cells. Moreover, we also observed that the apoptosis was significantly promoted in Ad‐CAIX^promotor^‐AIM2 ‐infected cells compared with Ad‐CAIX^promoter^‐infected cells (Figure 3E–G). We also examined the role of Ad‐CAIX^promotor^‐AIM2 on the migration of renal cancer cells through the transwell assays. The markedly decreased migration percentages were observed in Ad‐CAIX^promotor^‐AIM2‐infected 786‐O or OSRC‐2 cells in contrast to control cells (Fig. S1). Taken together, these results indicated that Ad‐CAIX^promotor^‐AIM2 could efficiently enhance the oncolytic effect by inhibiting renal cancer cell proliferation and increasing cell apoptosis.

### 3.3 The inflammasome activation was required for enhanced oncolytic effect of Ad‐CAIX^promotor^–AIM2

**Figure 4:**
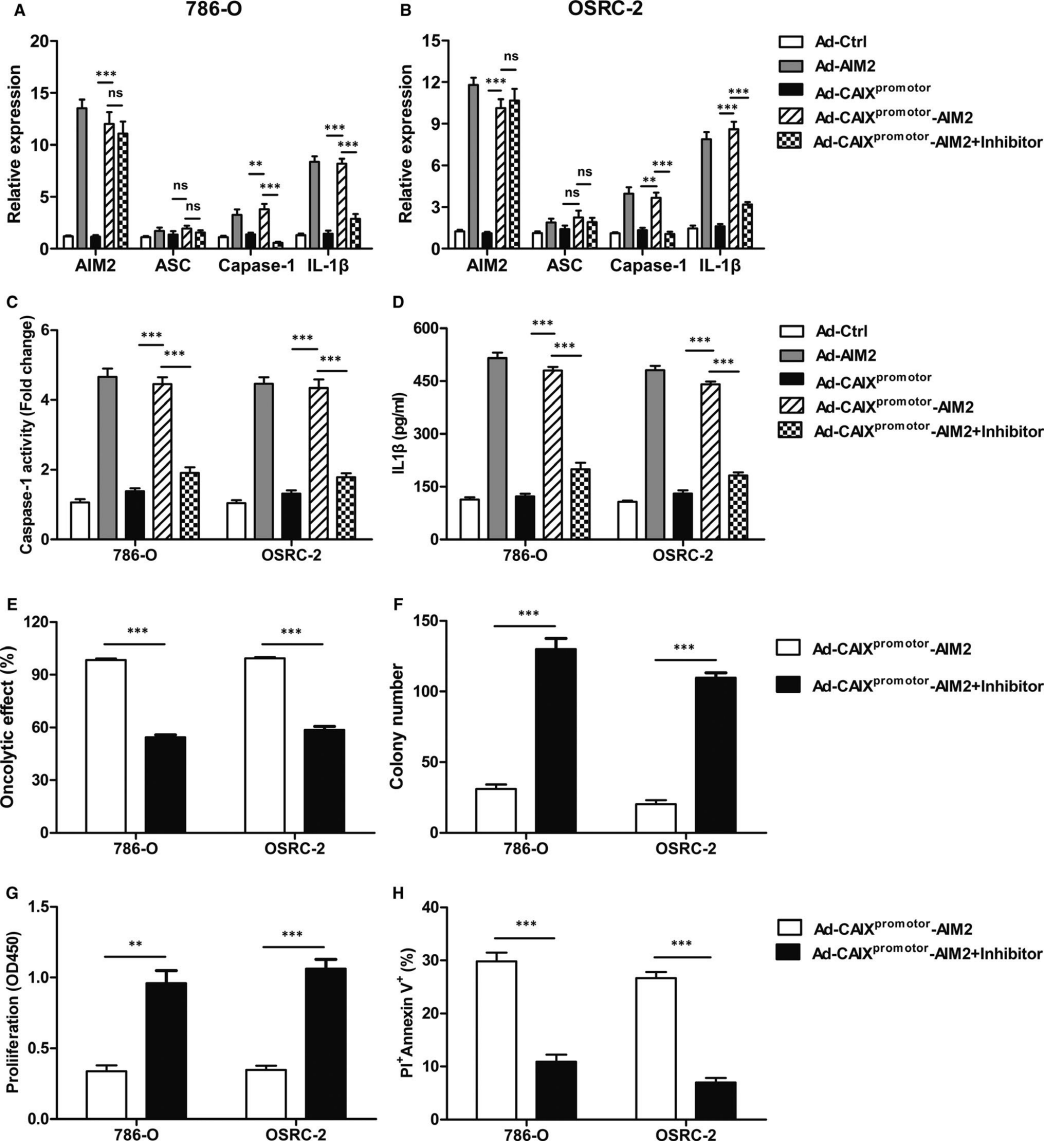
Ad‐CAIX^promotor^‐AIM2 suppressed the growth of renal cancer cells through enhanced the inflammasome activation. 786‐O or OSRC‐2 cells were infected with Ad‐CAIX^promotor^‐AIM2 or control followed with or without the AC‐YVAD‐CMK (50 μmol/L). (A and B) The levels of AIM2, ASC, caspase‐1 and IL‐1β were measured by Real‐time PCR in Ad‐CAIX^promotor^‐AIM2 or control‐infected 786‐O or OSRC‐2 cell lines. (C) The levels of caspase‐1 were measured in Ad‐CAIX^promotor^‐AIM2 or control‐infected renal cancer cells. (D) The levels of IL‐1β were measured by ELISA in Ad‐CAIX^promotor^‐AIM2 or control‐treated renal cancer cells. (E) The analysis of oncolytic effect in renal cancer cells. (F) Clone formation assay was performed to measure the cell proliferation in renal cancer cells. (G) The proliferation was detected by CCK‐8 assay. (H). The apoptosis was detected by flow cytometry in renal cancer cells. Data are from one representative experiment of three performed and presented as the mean ± SD. The different significance was set at *P < .05, **P < .01 and ***P < .001

Recent evidence suggests that AIM2 inflammasome activation plays an important role in suppressing the progression of tumour cells.^24^, ^25^ To further validate our hypothesis that Ad‐CAIX^promotor^‐AIM2 suppressed the growth of renal cancer cells by enhancing the inflammasome activation, 786‐O or OSRC‐2 was infected with Ad‐CAIX^promoter^‐AIM2 or control Ads followed with or without the downstream inflammatory caspase inhibitor YVAD‐CMK. Compared with control group, the expression of inflammasome components (AIM2, caspase‐1 and IL‐1β) (Figure 4A,B), caspase‐1 activation (Figure 4C) and the protein levels of IL‐1β (Figure 4D) were robustly elevated in Ad‐CAIX^promotor^‐AIM2 group, and suggested that the inflammasome was activated in Ad‐CAIX^promotor^–AIM2‐infected cells. The functional effect of AIM2‐mediated inflammasome activation was inhibited by YVAD‐CMK inflammasome inhibitor in Ad‐AIM2 or Ad‐CAIX^promotor^‐AIM2‐infected cells, and the levels of caspase‐1 and IL‐1β remarkably were reduced (Figure 4A‐D).

Accordingly, the inhibition of oncolytic ability and cell proliferation was prevented in YVAD‐CMK‐treated Ad‐CAIX^promotor^‐AIM2 group as contrasted to the Ad‐CAIX^promotor^‐AIM2 group (Figure 4E‐G). Furthermore, the enhancement of cell apoptosis by Ad‐CAIX^promotor^‐AIM2 also suppressed in YVAD‐CMK‐treated Ad‐CAIX^promotor^‐AIM2 cells (Figure 4H). The inhibition ability of cell migration by Ad‐CAIX^promotor^‐AIM2 in renal cancer cells was blocked by the YVAD‐CMK inhibitor (Fig. S2). These data indicated that the oncolytic efficacy of Ad‐CAIX^promotor^‐AIM2 is depended on the inflammasome activation.

### 3.4 The anti‐tumour effect by Ad‐CAIX^promoter^‐AIM2 was increased in OSRC‐2‐ xenograft mice

**Figure 5:**
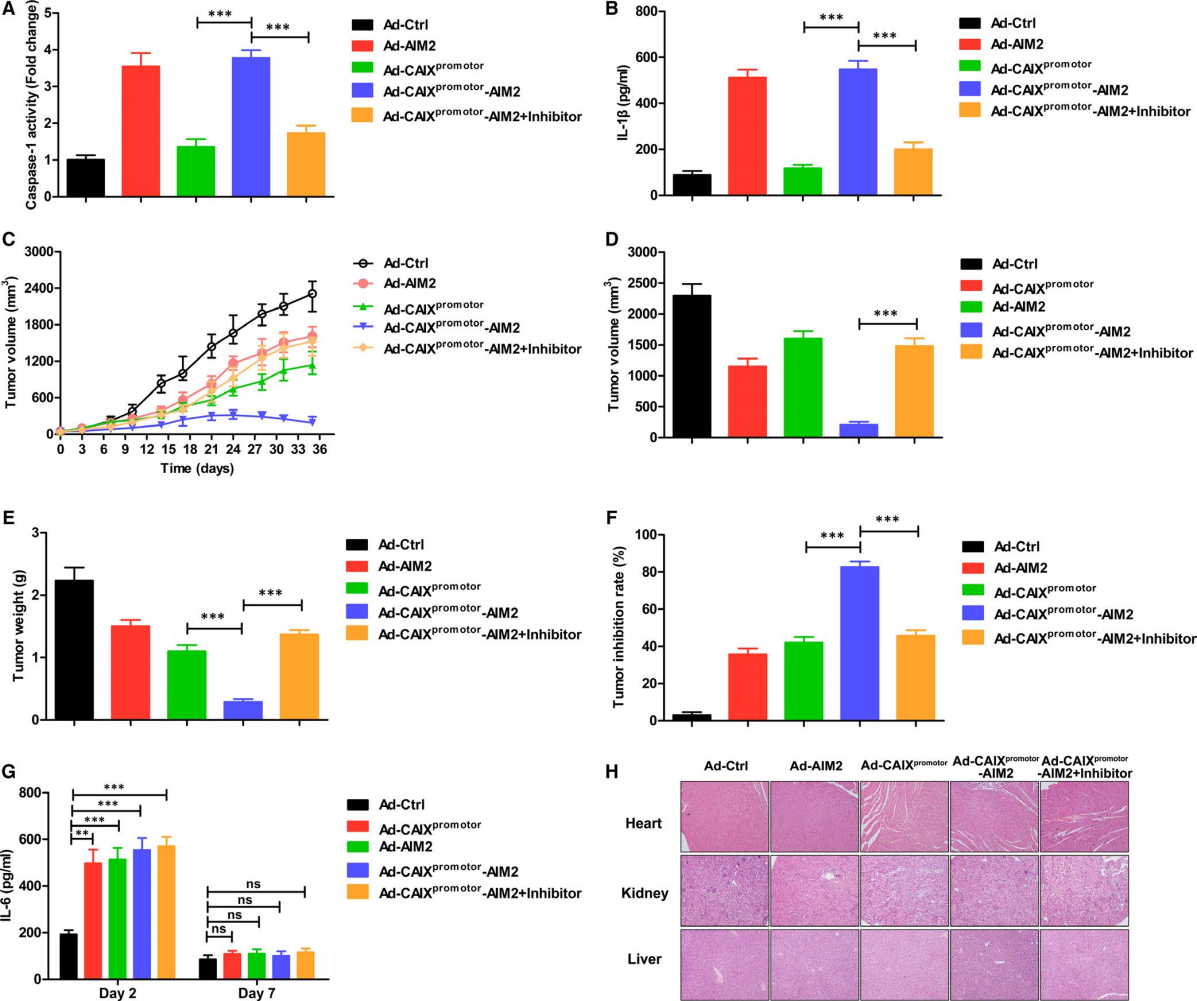
Ad‐CAIX^promotor^‐AIM2 inhibited the tumour growth of OSRC‐2 cell‐xenografted nude mice. The OSRC‐2‐xenografted nude mice were injected intratumourally Ad‐CAIX^promotor^‐AIM2 or control with or without inhibitor on tumour reached about 50 mm^3^ in volume. (A) Caspase‐1 activity was detected in tumour tissues. (B) The levels of IL‐1β were examined in tumour tissues. (C) Tumour progression of OSRC‐2‐xenograft was evaluated by measurement of tumour volume twice a week from day 0 to day 35. (D) Tumour volumes were measured at day 35 after tumour inoculation. (E) The tumour weights were monitored at day 35 after tumour inoculation. (F) Tumour inhibition rate. (G) At day 2 or 7 after the initial administration, the serum were collected, the levels of IL‐6 were determined by ELISA assay. (H) At day 35 after tumour inoculation, the pathology was evaluated by H&E staining of heart, kidney or liver tissues. Each experiment was performed independently at least three times and the results of one representative experiment are shown. Data are means ± SD, *P < .05, **P < .01 and ***P < .001; ns, no significant difference

To investigate the therapeutic effect of Ad‐CAIX^promotor^‐AIM2 treatment on tumour growth in vivo, OSRC‐2 cells were injected subcutaneously into nude mice. After 35 days, all of the mice were killed and tumours were resected. We firstly detected the inflammasome activation in tumour from various Ads‐treated OSRC‐2 xenograft mice. After blocking the effect of inflammasome activation by administration of YVAD‐CMK in vivo, the levels of capase‐1 and IL‐1β remarkably were reduced in tumour from Ad‐CAIX^promotor^‐AIM2 group than Ad‐CAIX^promotor^ group (Figure 5A,B). In Ad‐Ctrl group, tumours displayed rapid and continued outgrowth during the course of the experiment, and the tumour volume and weight were significantly inhibited in Ad‐CAIX^promotor^‐AIM2 treatment group (Figure 5C‐E). Tumour inhibition rate from animals treated with Ad‐CAIX^promotor^‐AIM2 exhibited a significant increase (Figure 5F). Furthermore, the rescued malignant behaviours including tumour volume and tumour weight were observed in YVAD‐CMK‐treated Ad‐CAIX^promotor^‐AIM2 group compared with Ad‐CAIX^promotor^‐AIM2 group (Figure 5C‐E). Moreover, Ad‐CAIX^promotor^‐AIM2 could not significantly elevate the level of cytokines, like IL‐6 in serum (Figure 5G). There is a non‐specific immune response demonstrated by normal pathological tissue of heart, liver and kidney in Ad‐CAIX^promotor^ ‐AIM2‐treated mice (Figure 5H), and indicated its low toxicity. Taken together, these data indicated that Ad‐CAIX^promotor^‐AIM2 treatment with low toxicity could effectively prevent the aggravation of tumour in renal cancer model.

### 3.5 Ad‐CAIX^promotor^‐AIM2 promoted the inhibition of lung metastasis in OSRC‐2‐lung metastasis model

**Figure 6:**
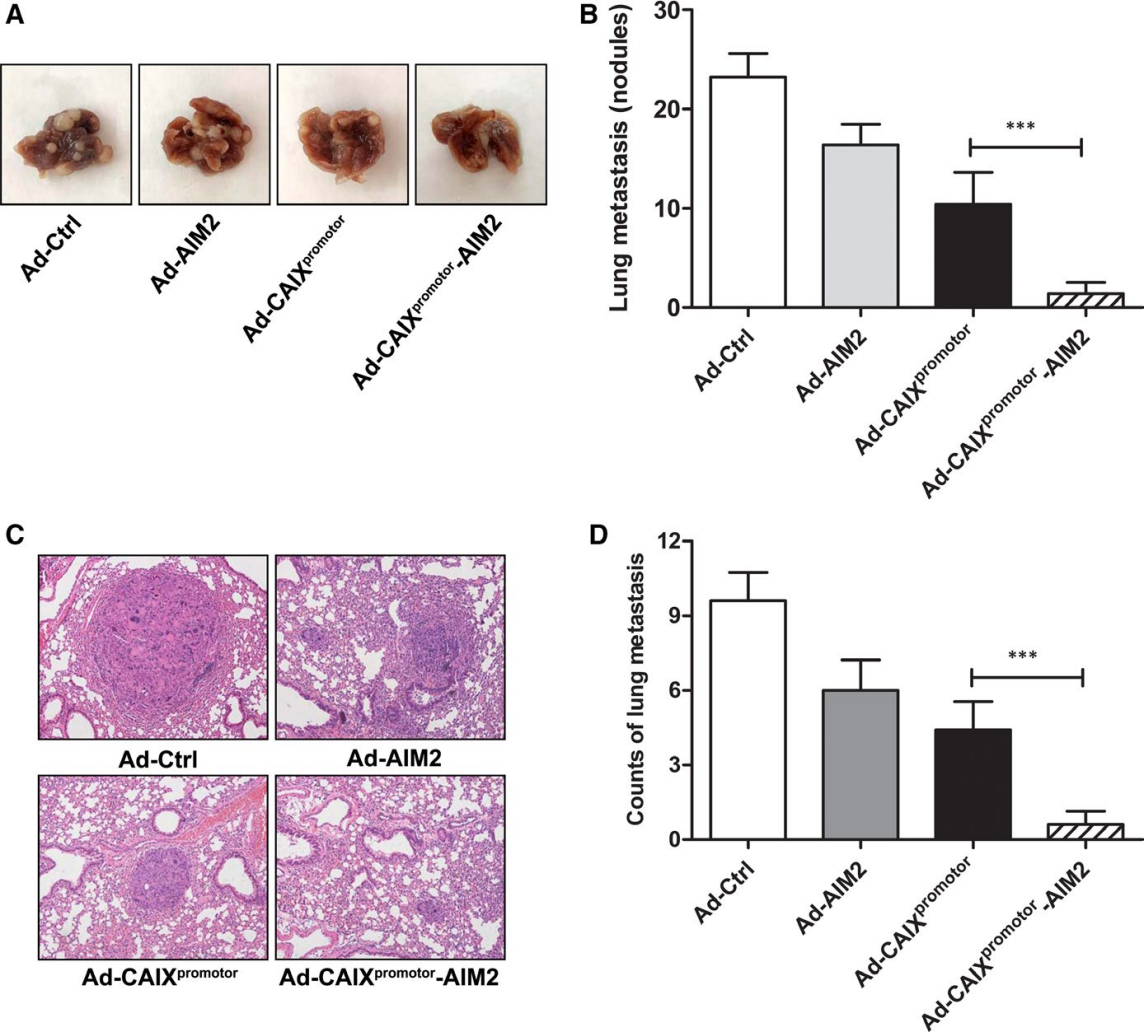
Therapeutic effects of Ad‐CAIX^promotor^‐AIM2 in lung metastasis model. (A) Representative view of lung tumours excised from renal cancer lung metastasis mice on day 28. (B) The numbers of metastatic nodules were quantified in the lungs of the tumour‐bearing mice. (C) Representative images of H&E staining for lung tissues; Scale bars: ×100 magnification. (D) The quantification of metastatic foci in the lung tissue. Each experiment was performed independently at least three times, and the results of one representative experiment are shown. Data are means ± SD, *P < .05, **P < .01 and ***P < .001

To assess whether Ad‐CAIX^promotor^‐AIM2 could also protect mice from tumour metastasis, we established a murine lung metastasis model of renal cancer. After OSRC‐2 cell inoculation, mice were intravenously injected with Ad‐Ctrl, Ad‐AIM2, Ad‐CAIX^promotor^ or Ad‐CAIX^promotor^‐AIM2. Mice were killed and lungs were removed on day 28 after tumour inoculation, and the visible metastases were counted. Ad‐CAIX^promotor^‐AIM2 treatment dramatically reduced the number of lung metastatic nodules compared with the Ad‐CAIX^promotor^ treatment (Figure 6A,B). The protective efficacy of Ad‐CAIX^promotor^‐AIM2 was further confirmed by and the quantification of metastatic foci from H&E staining of lung tissues of lung metastasis model (Figure 6C,D). These results indicated that Ad‐CAIX^promotor^‐AIM2 could suppress tumour lung metastasis of renal cancer.

## 4 DISCUSSION

CRAds can selectively replicate within malignant tumour cells resulting in cell lysis, which are considered as the effective therapeutic agents for of cancer.^7^ Their ability is based upon the tumour‐specific promotors to control viral regulatory genes and restrict the replication of CRAds in tumour cells.^26^ In this study, we explored that the therapeutic gene AIM2 enhanced the anti‐tumour effect of CAIX^promotor^ by regulating Ad tumour‐specific replication in renal cancer. Our results showed Ad‐CAIX^promotor^‐AIM2 treatment could increase oncolytic efficiency in renal cancer cells and exert the anti‐tumour effect in primary solid or metastasis tumour.

Previous study demonstrated that different extent expression of CAIX was observed in the human renal carcinoma cell lines, but not in normal cells, and indicated that CAIX can serve as a potential therapeutic target. The replication and expression of the CAIX^promotor^‐based Ads were observed in renal cancer cells, while not observed in ACHN cells and normal renal tubular epithelial cells HK‐2, which was proved the security of oncolytic adenovirus.^1^ In this study, the expression of CAIX was further confirmed and was significantly increased in Ketr‐3, 786‐O or OSRC‐2 cells (Figure 1A), which was consistent with the previous study. Therefore, CAIX in renal cancer shows apparent tumour specificity and its promotor could serve as an ideal target to construct tumour‐specific Ad for renal carcinoma treatment. CAIX^promotor^ was highly activated in human renal cancer cell lines and showed some effective treatment on renal carcinoma. However, Ad‐CAIX^promotor^ based on its therapeutic effect and potential side‐effects is still not satisfied on advanced renal carcinoma. To enhance the therapeutic effect of Ad‐CAIX^promotor^, novel Ads strategies are urgently needed to be developed for renal carcinoma treatment.

The function of Ads depends on the replication in tumour cells and killing them through the lytic replication cycle,^27^ and this effect can be enhanced by expressing anti‐cancer therapeutic molecules.^28^ Recent studies have demonstrated that this strategy is success by armed with tumour suppress gene to eradicate xenograft.^29^, ^30^ AIM2 as a tumour suppressor could inhibit the malignant behaviour of renal cancer cells, implied that AIM2 is a potent target gene for renal cell carcinoma therapy.^21^, ^22^ Here, AIM2 was selected as the target gene for Ad assembling to enhance the efficiency of anti‐tumour by inducing cell apoptosis and inhibiting tumour angiogenesis. Therefore, we developed the Ad‐CAIX^promotor^‐AIM2 that was an E1B55kD deletion with the E1A gene controlled by CAIX^promotor^, armed with the AIM2 gene to enhance the therapeutic efficacy of renal cancer.

The cytotoxicity or potent therapeutic efficacy against renal carcinoma cells was evaluated in vitro. Ad‐CAIX^promotor^‐AIM2 demonstrates its tumour cell‐specific replication by efficiently expressing E1A and AIM2 in renal carcinoma cells. Compared with the Ad‐CAIX^promotor^, the significantly enhanced cytotoxicity was seen in infected‐786‐O or OSRC cells. Similarly, Ad‐CAIX^promotor^‐AIM2 remarkably enhanced the inhibition of cell proliferation and migration and promoted cell apoptosis in infected renal cancer cells. Moreover, the inflammasome signalling can prime natural killer (NK) cells through IL‐1β or IL‐18 and exert anti‐cancer effects by specialized programmed cell death called pyroptosis and immune regulatory functions.^31^, ^32^ Previous study showed that Akt is a pivotal serine/threonine kinase and promotes epithelial‐mesenchymal transition process through NF‐κB and GSK‐3β in cell biological progression including cell proliferation, apoptosis and metabolism. AIM2 inflammasome activation impaired cell invasion and metastasis via inhibiting Akt pathway in colorectal cancer.^33^ Furthermore, overexpression of AIM2 in human renal cancer suppressed cell metastasis via enhancing autophagy induction.^21^ Our results indicated that inflammasome inhibitor YVAD‐CMK resulted in the reduction of oncolytic effect by Ad‐CAIX^promotor^‐AIM2, implied that this efficiency was dependent on AIM2 inflammasome activation responses.

To further explore the therapeutic effect of Ad‐CAIX^promotor^‐AIM2, OSRC‐2 xenograft mice were injected subcutaneously into nude mice. The tumour growth inhibition curve accompanied by inflammasome activation in tumour and indicated that the oncolytic adenovirus could effectively inhibit the growth of primary solid tumour. This effect by AIM2 inflammasome signal was confirmed by blocking of inflammasome activation by VAD‐CMK in vivo. However, the xenograft could not be eliminated totally only by Ad‐CAIX^promotor^‐AIM2, which might be due to immune escape from the tumour, such as the expression of PD‐L1 inhibitory signals or immunosuppressive cells. In future study, we would further investigate that the potent way is combination of the Ad with blocking of PD‐L1 or immunosuppressive cells to completely eliminate tumour cells in tumour microenvironment. Of note, the Ad expressed AIM2, which was under the control of CAIX^promotor^, thus ensuring no harm was done to the normal surrounding cells. Our results showed that Ad‐CAIX^promotor^‐AIM2 could not significantly elevate the level of IL‐6 cytokine in serum and reduce the bodyweight, and indicated that there is a non‐specific immune response and low toxicity.

In conclusion, a novel Ad was engineered with the E1A gene controlled by the ‐CAIX^promotor^ and AIM2 gene. Ad‐CAIX^promotor^‐AIM2 showed the enhanced oncolysis in CAIX‐positive renal cancer cells compared with the control. Ad‐CAIX^promotor^‐AIM2 indicated the potent growth inhibition effects on renal cancer models of primary or lung metastasis. The therapeutic strategy of AIM2‐based Ads might be a potential and promising approach for the therapy of primary solid or metastasis tumours.

## CONFLICT OF INTEREST

The authors declare no conflict of interest.

## AUTHOR CONTRIBUTION

**Dafei Chai:** Conceptualization (equal); Data curation (equal); Formal analysis (equal); Funding acquisition (equal); Investigation (equal); Writing‐original draft (equal). **Dong Qiu:** Investigation (equal); Methodology (equal). **Zichun Zhang:** Investigation (equal); Methodology (equal). **Shang Yuchen Shi:** Data curation (equal); Investigation (equal); Methodology (equal). **Gang Wang:** Conceptualization (equal); Funding acquisition (equal); Resources (equal). **Lin Fang:** Conceptualization (equal); Resources (equal). **Huizhong Li:** Methodology (equal); Resources (equal). **Hailong Li:** Funding acquisition (equal); Methodology (equal); Resources (equal). **Hui Tian:** Methodology (equal); Resources (equal). **Junnian Zheng:** Conceptualization (equal); Funding acquisition (equal); Project administration (equal); Resources (equal); Supervision (equal); Writing‐review & editing (equal).

## Supporting information

Supplementary Material

Fig S1

Fig S2

## Data Availability

The data supporting the findings of this study are included within the article.
