# Supplementary material for: Absent in melanoma 2 enhances anti‐tumour effects of CAIX promotor controlled conditionally replicative adenovirus in renal cancer
Source: J Cell Mol Med. 2020 Jul 29;24(18):10744–55. doi: 10.1111/jcmm.15697 (PMC7521288; doi:10.1111/jcmm.15697)
Supplement: Supplementary file 1 — Supplementary Material [file JCMM-24-10744-s001.doc]

**Supplementary Methods**

**Cell migration assay**

Transwell assay was performed to determine cell migration in 2-chamber plates with a pore size of 8 μm. The upper chambers of 24-well plates were added 786-O and OS-RC-2 cells at a density of 5 × 10^4^ cells per well in 200 μl serum free medium. The lower chambers was added 600 μl complete medium. After incubation for 16 h, the cells that had passed through the membrane were fixed in methanol, stained with crystal violet. Non-traversed cells were carefully removed from the upper surface of the filter, and traversed cells on the lower side of the filter were counted.

**Supplementary Figure legends**

**Figure 3S. Ad-CAIX^promotor^-AIM2 increased the suppression of renal cancer cell migration.** (A). The present images of cell migration in 786-O or OSRC-2 cells infected with Ad-Ctrl, Ad-AIM2, Ad-CAIX^promotor^ or Ad-CAIX^promotor^-AIM2. (B). Statistical histograms of migrated cell percentages among various Ads in 786-O or OSRC-2 cells, respectively. Data are from one representative experiment of three performed and presented as the mean ±SD. The different significance was set at ***p < 0.001.

Figure 4S. **The enhanced suppression of cell migration by Ad-CAIX^promotor^-AIM2 was relieved by YVAD-CMK treatment**. 786-O or OSRC-2 cells were infected with Ad-CAIX^promotor^-AIM2 or control followed with or without the AC-YVAD-CMK (50 μmol/L). The percentages of migrated cells were analyzed among various Ads infected 786-O or OSRC-2 cells, respectively. Each experiment was performed independently at least three times. Data are means ± SD, ***p<0.001.
