# Supplementary figures and images for: Absent in melanoma 2 enhances anti‐tumour effects of CAIX promotor controlled conditionally replicative adenovirus in renal cancer
Source: J Cell Mol Med. 2020 Jul 29;24(18):10744–55. doi: 10.1111/jcmm.15697 (PMC7521288; doi:10.1111/jcmm.15697)

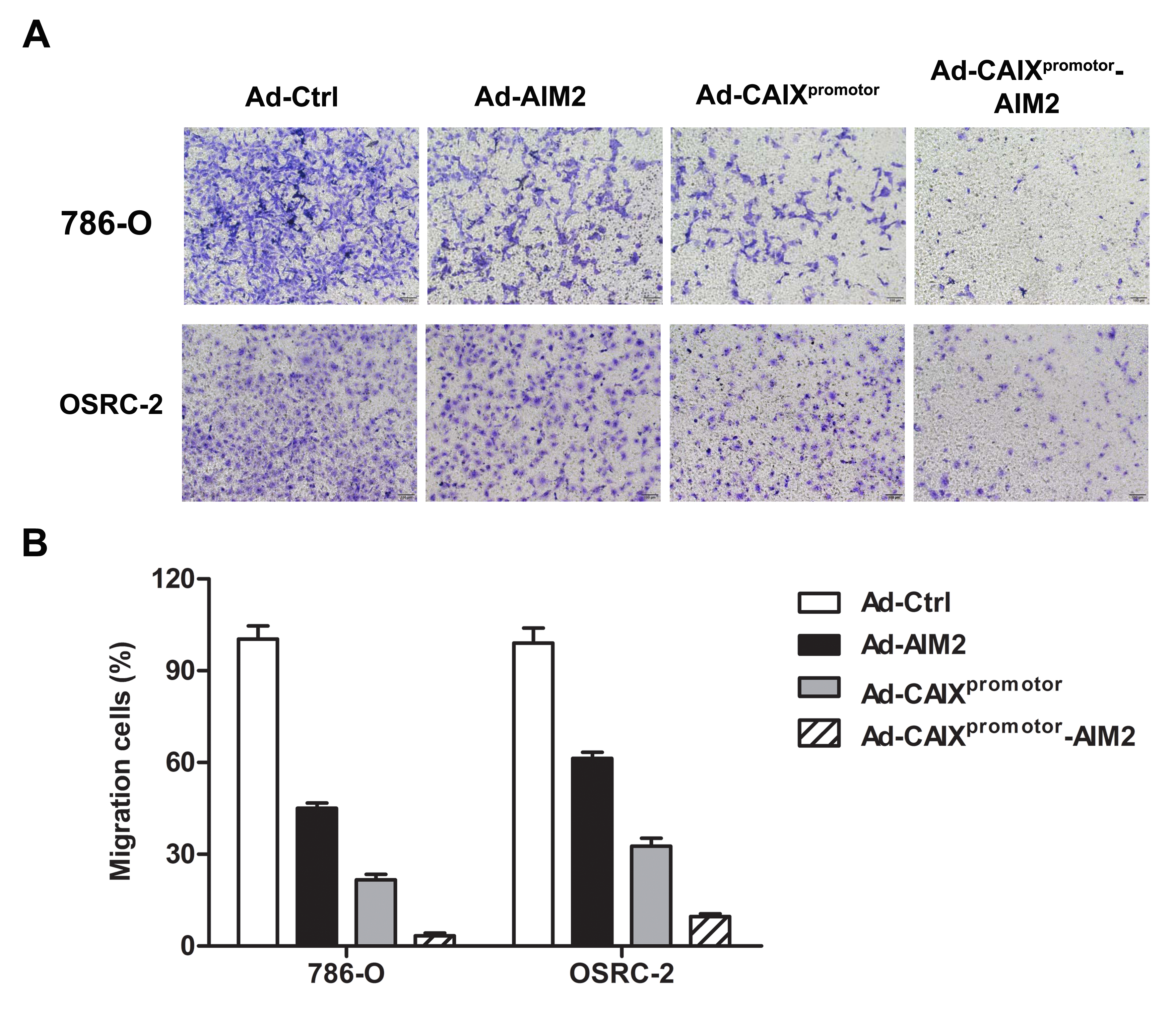

Supplement: Supplementary file 2 — Fig S1 [file JCMM-24-10744-s002.tif]

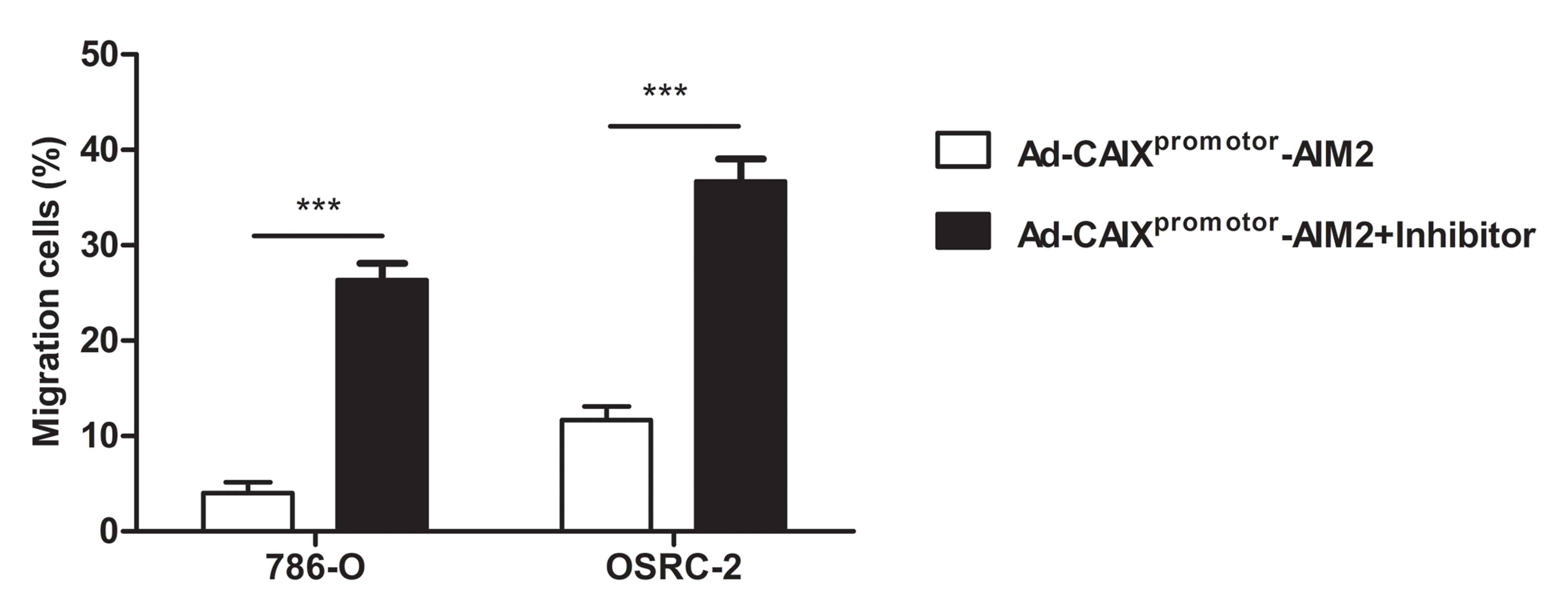

Supplement: Supplementary file 3 — Fig S2 [file JCMM-24-10744-s003.tif]
